# Establishment of molecular genetic approaches to study gene expression and function in an invasive hemipteran, *Halyomorpha halys*

**DOI:** 10.1186/s13227-017-0078-6

**Published:** 2017-10-18

**Authors:** Yong Lu, Mengyao Chen, Katie Reding, Leslie Pick

**Affiliations:** 1 Department of Entomology, University of Maryland, College Park, MD 20742 USA; 2 Present Address: Department of Anesthesiology, Stony Brook Medicine, 101 Nicolls Rd, Stony Brook, NY 11794 USA

**Keywords:** Brown Marmorated Stink Bug, *H. halys*, RNAi, Homeotic, Hemiptera, *Engrailed*, *Scr*, *Even*-*skipped*

## Abstract

Hemiptera is a large clade of insects understudied in terms of developmental biology. *Halyomorpha halys*, the Brown Marmorated Stink Bug (BMSB, referred to throughout as *H. halys*), is an invasive hemipteran pest of the mid-Atlantic region of the USA that has rapidly spread to other regions in recent years, devastating a wide range of crops using a piercing and sucking mechanism. Its phylogenetic position, polyphagous habits, and rapid spread in the USA suggested that *H. halys* would be an ideal system to broaden our knowledge of developmental mechanisms in insects. We and others previously generated transcriptome sequences from different life stages of this insect. Here, we describe tools to examine gene expression patterns in whole-mount *H. halys* embryos and to test the response of *H. halys* to RNA interference (RNAi). We show that spatial and temporal patterns of gene expression in *H. halys* can be effectively monitored by both immunostaining and in situ hybridization. We also show that delivery of dsRNA to adult females knocks down gene function in offspring, using the homeotic gene *Sex combs reduced* (*Scr*). Knockdown of *Hh*-*Scr* resulted in dramatic malformations of the mouthparts, demonstrating for the first time that RNAi is effective in this species. Our results suggest that, despite difficulties with long-term laboratory culture of *H. halys*, this species shows promise as a developmental system.

**Electronic supplementary material:**

The online version of this article (doi:10.1186/s13227-017-0078-6) contains supplementary material, which is available to authorized users.

## Background

Most studies of arthropod evo-devo have utilized holometabolous insects, particularly *Drosophila melanogaster*, and, more recently, *Tribolium castaneum* (*T. castaneum*) which are well-developed laboratory model systems with a wide array of resources available for communities of researchers [1–7]. The order Hemiptera, which includes at least 54 families of the true bugs (Heteroptera), is the largest non-holometabolous insect order with more than 80,000 described species [8, 9]. Hemiptera are hemimetabolous insects, sharing piercing and sucking mouth parts that cause harm to both plants and animals, by virtue of direct physical damage, as well as by transmission of pathogens. The order includes major agricultural pests, such as aphids, stink bugs, and white flies, as well as pests of humans, such as kissing bugs, vectors of serious human disease (reviewed in [10, 11]). As is the case for hemimetabolous taxa in general, studies of hemipteran development have been underrepresented in the evo-devo field to date, limiting our ability to probe the origins of diversity within insects. Within the order Hemiptera, the milkweed bug *Oncopeltus fasciatus* (*O. fasciatus*, Hemiptera: Lygaeidae) is emerging as a model species (see, for examples, [12–18]). However, additional systems are required for comparative studies within the hemimetabolous insects, to understand both general rules controlling the development of insects that do not undergo metamorphosis, and to understand the developmental basis of novelties, including for example, the large differences in host preference (plant feeders vs. human hosts), color patterns, and habitat choice seen within this clade.

With an estimated divergence from *O. fasciatus* of 244 MYA [19], *Halyomorpha halys*, commonly known as the Brown Marmorated Stink Bug (*H. halys*, Hemiptera: Pentatomidae), represents a distant branch within Hemiptera, providing a useful phylogenetic point for comparative studies to *O. fasciatus*. *H. halys* is a polyphagous insect which feeds on tree fruits, vegetables, legumes, and ornamentals in the field and in nursery crops (reviewed in [20, 21]), as opposed to *O. fasciatus*, which is a highly specialized feeder. Another justification for studying *H. halys* is that it is a serious agricultural pest, which has caused significant damage in the Mid-Atlantic region of the USA in recent years [22, 23]. Studies of gene expression and gene function, as well as the ability to manipulate genes, will uncover information about the basic biology of *H. halys*, while providing novel means to make use of genetic approaches for pest control.

Here, we report the first molecular methods for *H. halys*. We have developed methods to examine spatiotemporal gene expression, using segmentation genes *even*-*skipped* (*eve*) and *engrailed* (*en*) for in situ hybridization. Using a cross-reactive antibody for En, we also provide methods for immunohistochemistry in *H. halys* embryos. Based upon the dramatic homeotic transformation of proboscis to leg seen after RNAi-mediated knockdown of the *Sex combs reduced* gene (*Scr*) in *O. fasciatus*, and in a cockroach [15, 24, 25], we used this gene to test whether *H. halys* are capable of systemic responses to injected dsRNA. We found that injection of *Scr* dsRNA into adult *H. halys* females resulted in malformation of the mouthparts in their offspring, suggestive of homeotic transformation toward leg. These results provide a starting point for comparative evolutionary developmental studies in a thus far understudied hemipteran species, while also suggesting that RNAi can be an effective strategy to control *H. halys* pests.

## Methods

### Insect husbandry and embryo collection

Laboratory colonies of *H. halys* were initially reared as previously described [26]. Briefly, *H. halys* were collected in soybean fields at the University of Maryland Beltsville Research Farm. The collected *H. halys* were kept in mesh cages (60 × 30 × 35 cm). Potted green bean plants (*Phaseolus vulgaris*) were the major food source and hiding place for the bugs. Organic green bean pods and raw sunflower seeds were added to the cages to provide extra nourishment. These foods were replaced with fresh ones once or twice a week. All foods were certified organic and were washed extensively before placement in cages. Other diet supplements we tested included blueberries, apples, grapes, and carrots. We did not notice any difference in *H. halys* growth with these extra food sources. After several colony collapses using this approach, we switched to a rearing protocol kindly provided to us by Dr. Don Weber [27]. We grew a new colony from ten egg masses provided by Dr. Weber’s laboratory. Different generations were kept separately, in order to track the health of our colony and keep the most reproductive individuals together. Briefly, eggs and first instar nymphs were kept in small petri dishes (5.5 cm in diameter) with wet cotton and pieces of fresh organic green beans. When nymphs reached the second instar, generally after 5 days, they were moved to clean plastic cylindrical containers (18.5 cm in diameter × 20.5 cm in height) with fresh organic green bean pods, sunflower and buckwheat seeds, and wet cotton. A piece of fine plastic mesh screen (32 × 32 mesh per inch) was also added to each adult cage as an egg-laying substrate. While *H. halys* egg masses stick to most surfaces fairly well, they can be easily peeled off of this plastic mesh. All *H. halys* cages were kept at 25 °C, RH of 55 ± 5%, with a 16 h light:8 h dark photoperiod. Every other day, the green beans were replaced in all cages and petri dishes. At this time, egg masses and any dead adults were removed from adult cages; the numbers of clutches and dead males and females were recorded so that cage population and female fecundity could be tracked. For timed egg collections, cages were checked every 4 h for newly laid eggs. The eggs were removed from the cages and kept in petri dishes under the same environmental conditions described above until the desired time points were reached. Under these conditions, the overall life cycle (egg to fertile adult) was ~ 1.5 months, with eggs hatching to the first nymphal stage in 5 days and each subsequent instar lasting between 5 and 10 days.

### Identification of genes of interest

Prior to availability of genome or transcriptome data, degenerate PCR was carried out to isolate regions of *H. halys* orthologs of *engrailed* (*Hh*-*en*) and *Sex combs reduced* (*Hh*-*Scr*). Primers were: *Hh*-*en*: DEGN*en*F 5′-GARAAYMGNTAYYTNACNGA-3′ and DEG*en*R 5′-RTGRTTRTANARNCCYTGNGC-3′; ScrdegF 5′-CCRCARATHTAYCCRTGGATG-3′ and ScrdegR1 5′-CATRTGGYANGGNACRATRTTCAT-3′. Sequences were verified by comparison with transcriptome data. Assembled *H. halys* RNA-seq data [28] in FASTA format were used to create a local *H. halys* BLAST database using the BLAST + package [29]. BLAST searches were carried out using the sequence of products generated by degenerate PCR, followed by TBLASTN using full-length *D. melanogaster* En or Scr protein sequences as the query sequences. To identify *H. halys even*-*skipped* (*eve*), full-length *D. melanogaster* Eve was used as query with the local *H. halys* BLAST database as the subject database. Reciprocal BLAST with the insect non-redundant protein sequence database was carried out to find orthologs. Predicted *H. halys* genes were experimentally verified by reverse transcription PCR (RT-PCR) followed by Sanger sequencing. Gene accessions: *Hh*-*eve*, GenBank: GBHT01012779.1; *Hh*-*en*, GenBank: GBHT01012041.1; *Hh*-*Scr*, GenBank: GBHT01003272.1 [28].

### Embryo fixation

To collect embryos, the plastic mesh egg-laying substrate was removed from the cage and embryos were simply peeled off the mesh and dropped into 2-ml centrifuge tubes, with ~ 20 embryos per tube. Embryos for in situ hybridization and immunostaining were aged to 18–72 h after egg laying (AEL). The fixation protocol was modified from that developed for *O. fasciatus*, kindly shared by Dr. Ariel Chipman’s laboratory [30]. In brief, 600 μl of water was added to each tube of embryos which was placed in boiling water for 3 min and then placed on ice for 6 min. After the water was removed, 600 μl of heptane and 600 μl 4% paraformaldehyde (PFA) in PBS (0.137 M NaCl, 0.0027 M KCl, 0.0015 M KH_2_PO_4_, 0.008 M Na_2_HPO_4_) were added. Gentle shaking brought the embryos to the interface. Tubes were shaken vigorously on a Vortex mixer for 20 min. After shaking, the heptane and PFA were removed, and the embryos were rinsed once with heptane, then once with methanol. The embryos together with methanol were then put into wells on depression concave slides and the eggshells, which are rather thick in this species, were manually removed with forceps under a dissection microscope. The embryos were then passed through 75, 50, and 25% methanol/PBST (phosphate-buffered saline, 0.05% Tween^®^ 20) gradient rinses for rehydration. The rehydrated embryos were fixed with 4% PFA in PBST for 90 min on a nutator. The fixed embryos were then washed three times with methanol and stored in methanol at − 20 °C for future use.

### Immunohistochemistry

Embryos were collected and fixed as described above, removed from − 20 °C, and passed through a 75, 50, and 25% methanol/PBST gradient for rehydration. Embryos were then rocked on a nutator in 5% BSA in PBST for 2–3 h to block non-specific binding. After blocking, the embryos were incubated with a 1:10 dilution of monoclonal anti-Engrailed antibody 4D9 (Developmental Studies Hybridoma Bank) in 5% BSA at 4 °C overnight. The 4D9 antibody was removed, and the embryos were washed three times for 20 min each with PBST. The embryos were then incubated with 1:300 biotinylated goat anti-mouse IgG antibody (Vector Labs) for 2 h at room temperature. The secondary antibody was then removed, and embryos were washed with PBST three times for 20 min each. After washing, the embryos were incubated 1 h with ABC reagent (avidin–biotin complex, Vector Labs) followed by three 20-min washes with PBST. Detection by a color reaction was carried out using the SigmaFast DAB kit (Sigma-Aldrich). Expression was monitored under a dissection microscope and terminated when expression was evident, usually within 30 min. The DAB solution was then removed, and embryos were rinsed three times with PBS. Embryos were post-fixed with 4% PFA for 20 min rinsed with and transferred to PBST, and germband embryos were removed from the yolk using forceps. The post-fix was added because the yolk was very sticky, and the embryos were quite fragile and difficult to dissect. Adding methanol to PBST (1:1) was found to decrease the stickiness of the yolk and improve the dissections. Embryos were rehydrated in PBS and then transferred to 90% glycerol/PBS, where they were held overnight. Germ bands were mounted in 90% glycerol/PBS. Photographs were taken under a dissection microscope (Leica M420, 16–20×).

### Whole-mount in situ hybridization

Digoxygenin-labeled probes were made by in vitro transcription using PCR products as template, using the MEGAscript^®^ T7 Transcription Kit (Life Technologies) following the manufacturer’s recommendations. T7 promoter sequences were added to the reverse PCR primers for amplification of *H. halys* cDNA, generated from mixed-stage embryos. The primers used were: *Hh*-*enF* 5′-TACCCTTCTCCGTCGACAAC-3′ and *Hh*-*en*RT7 5′-TAATACGACTCACTATAGGGAGACGGCCTCTTGTCTTCTTTGT-3′ generating a 252 bp fragment for *Hh-en; Hh-eve 2F* 5′-AGGAGCATGTCATCGAGAAGG-3′ and *Hh*-Eve2RT7 5′-TAATACGACTCACTATAGGGAGAACTATCTTCCTGCTATCACTGGT-3′ to amplify a 231-bp fragment including coding region and 3’UTR of *Hh.eve*. Embryos were fixed and rehydrated as described above. After rehydration, the embryos were pre-hybridized with hybridization buffer (50% formamide, 5 × SSC, 0.1% Tween 20, 50 μg/ml yeast tRNA, 5% dextran, 50 μg/ml heparin) at 55 °C for 1–4 h. After pre-hybridization, a 1:100 dilution of probe in hybridization buffer was added to the embryos and incubated at 55 °C overnight (16–18 h). The probe was then removed, and the embryos were washed twice with hybridization buffer at 55 °C for 15 min each, followed by two washes in 2 × SSC at room temperature for 30 min and one wash in 0.2 × SSC at room temperature for 30 min. The embryos were rinsed three times with PBST. After rinsing, the embryos were incubated with 10% sheep serum in PBST for 1–4 h at room temperature to block non-specific binding. Embryos were then incubated with anti-digoxygenin–AP antibody (1:1500, Roche) at 4 °C overnight. After incubation, the antibody was removed, and the embryos were washed five times in PBST for 20 min each. For detection, the embryos were washed with staining buffer (100 mM NaCl, 50 mM MgCl_2_, 100 mM Tris pH 9.5, 0.1% Tween 20) three times for 5 min each. Staining was carried out using NBT/BCIP solution (Roche) diluted in staining buffer. The embryos were checked under a dissection microscope every 10 min until the desired color reaction had developed, generally within 1 h. The color reaction was stopped by adding PBST to the staining solution. The embryos were then washed with PBST three times for 5 min each to remove the staining solution from the embryos. The embryos were washed with 50% methanol in PBST for 5 min and 100% methanol for another 5 min, and then placed in 100% ethanol for 30 min to 2 h. The embryos were washed with 50% methanol in PBST for 5 min and with PBST three times for 5 min each. Blastoderm stage embryos were transferred to depression slides to take pictures; germ band stage embryos were dissected out of yolk and mounted on slides with 90% glycerol. Photographs were taken under a dissection microscope (Leica M420, 16–20×).

### Double-strand RNA (dsRNA) synthesis

Primers were designed to amplify a 327-bp region of *Hh*-*Scr*, with T7 promoter sequences added to the 5′ end of both forward and reverse primers. Primer sequences: *Hh*-*Scr*FT7 5′-TAATACGACTCACTATAGGGAGAGCAGGACCTGACTACGTCCTC-3′ and *Hh*-*Scr*RT7 5′-AATACGACTCACTATAGGGAGATCCAGCTCCAGCGTCTGGTA-3′ (T7 promoter sequences underlined). PCR was carried out with cDNA that had been made from 0-to-6-day-old embryos using the manufacturer’s recommended standard conditions (Reverse Transcription system, Promega). The PCR products were purified and sent out for sequencing (Genewiz) to confirm that the correct gene was amplified. The purified PCR product (Gel Extraction Kit, Qiagen) was used as the template for in vitro transcription using the MEGAscript^®^ T7 Transcription Kit (Life Technologies) following the manufacturer’s recommendations. The final product was treated with DNase from the transcription kit to degrade the DNA template. In order to anneal the in vitro transcription product single-stranded RNAs, transcription products were heated to 94 °C for 5 min and slowly cooled in a PCR machine by decreasing the temperature 0.8 °C every minute until 45 °C was reached (TPersonal, Biometra). The annealed double-strand RNA was precipitated with 1/10 volume of 3 M sodium acetate (pH, 5.2) and 2× volume of ethanol and was then dissolved in 20–40 μl injection buffer (0.1 mM NaH_2_PO_4_, 5 mM KCl, pH 6.8), and stored at − 20 °C. The concentration of double-stranded RNA was measured with a NanoDrop spectrophotometer.

The dsRNA was injected into adult female *H. halys* using a Hamilton syringe and needle, as described previously for *O. fasciatus* [50]. *H. halys* females were anesthetized under CO_2_, and the Hamilton needle was inserted into the abdomen between the third and fourth abdominal sternites (Additional file 1: Figure 1). Each female *H. halys* was injected with 2–6 μl *Scr* dsRNA at a concentration of 3 μg/ul. Four-to-ten individual females were injected in each experiment. After injection, the needle was held at the injection site for approximately 1 min to prevent leakage from the injection site. Injected females were kept separately for 1 day to allow for recovery before 2–3 males were added to each cage for mating. Eggs were collected and allowed to hatch, and defects were assessed in both hatched nymphs and unhatched embryos.

## Results

### Gene expression in whole-mount *H. halys* embryos

*Hh*-*engrailed* (*Hh*-*en*) and *Hh*-*even*-*skipped* (*Hh*-*eve*) were isolated for this study because of their unique expression patterns and known functions in other insects, thereby serving as good candidate genes for the establishment of molecular staining techniques for *H. halys*. To isolate *Hh*-*en*, degenerate primers were used to amplify cDNA prepared from 1-to-5-day-old *H. halys* embryos. A sequence of 286 bp was acquired and used as the query in a BLASTN search against the *H. halys* transcriptome database and compared with TBLASTN results. One 566-bp sequence was shared by both BLASTN and TBLASTN results and encodes a 188 amino acid region, which includes the 60 amino acid homeodomain. The *Hh*-En homeodomain is 85% identical to *D. melanogaster* En, and 83% identical to *D. melanogaster* Inv. The *Hh*-En sequence does not have the RS-motif, which is found in Inv proteins [31, 32], suggesting it is more closely related to En (Additional file 2: Figure 2a). *Hh*-*eve* sequence was identified through BLAST searches of the transcriptome data using the full-length *D. melanogaster* Eve protein sequence as query and orthology was confirmed by reciprocal BLAST. The *Hh*-*eve* sequence isolated encodes 221 amino acids. The homeodomain is 90% identical to that of *D. melanogaster* Eve (Additional file 3: Figure 2b).

**Fig. 1:**
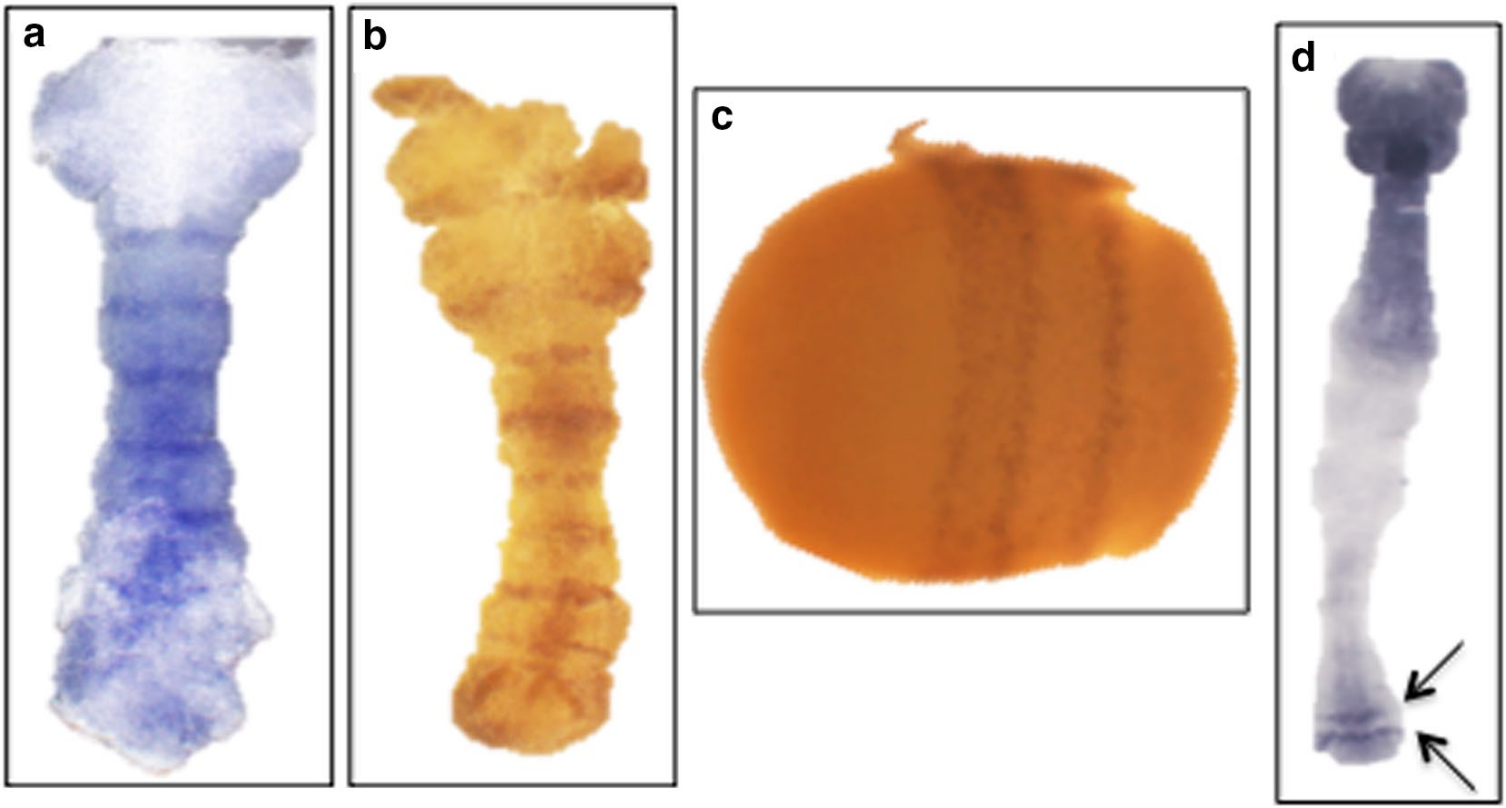
Gene expression in *H. halys* embryos. **a** In situ hybridization in *H. halys* germ band embryos using an *Hh*–*en* probe. Six stripes were detected. The anterior and posterior parts of the embryo remained covered with yolk in this photograph. **b** En antibody staining to an embryo slightly older than that shown in (**a**). Nine stripes were observed. **c** A blastoderm stage embryo expressing three *Hh*-*eve* stripes in the central region of the embryo. **d** A germ band with two *Hh*-*eve* stripes at or near the segment addition zone. (**a**, **b**, **d**) anterior (top) to posterior (bottom); (**c**) embryo orientated anterior (left), posterior (right)

In *D. melanogaster*, *en* is a segment polarity gene expressed in segmental stripes in the primordia of the posterior compartment of each segment [33, 34]. Similar segmental expression patterns for *en* were observed in a wide range of other species including all insects examined to date as well as more distant arthropods [35–38]. This high degree of conservation of expression makes *en* a useful marker for establishing techniques to monitor gene expression in embryos of diverse species, as it is expected to be expressed in clear, segmental stripes in early embryos of virtually any insect. This type of clear expression pattern allows one to distinguish false positive patterns from true patterns, as it is easy to tell which staining is background and which is a *bona fide* signal. Thus, we first characterized the expression of *Hh*-*en* in *H. halys* embryos using in situ hybridization protocols on early germ band stage *H. halys* embryos. As shown in Fig. 1a, *Hh*-*en* mRNA was detected by in situ hybridization in segmental stripes, as expected. To test whether in situ hybridization works for other genes expressed in early *H. halys* embryos, an ortholog of the *D. melanogaster* pair-rule gene (PRG) *eve* was isolated. In contrast to *en*, which is expressed segmentally throughout arthropods [36], orthologs of *D. melanogaster* PRGs vary in expression in different arthropod taxa (e.g., [39–43]). This in part reflects the fact that segments are specified more or less simultaneously at the blastoderm stage in *D. melanogaster*, reflecting its derived, long germ mode of development. In contrast, most other insects, including Hemiptera, specify only a small number of segments at blastoderm, with additional segments added sequentially from a growth or segment addition zone (short- or intermediate germ mode, reviewed in [44, 45]). Accordingly, *Dm*-*eve* is expressed in seven stripes in the *D. melanogaster* blastoderm in the primordia of segmental regions missing in *eve* mutants [46]. In contrast, in the hemipteran *O. fasciatus*, *eve* is expressed in the segment addition zone, with stripes emerging sequentially in elongating germ bands [47]. *Hh*-*eve* expression was detected in clear stripes in both blastoderm (Fig. 1c) and germ band stage (Fig. 1d) embryos. In blastoderm stage embryos, three *Hh*-*eve* stripes were detected in the central region of the embryo. This number of PRG stripes is suggestive of intermediate germ development, as only single *eve* stripes were observed at the blastoderm stage for short germ *T. castaneum*, while four *eve* stripes were observed for intermediate germ beetles [48, 49]. In elongating germband embryos, two *Hh*-*eve* stripes were detected at or just anterior to the segment addition zone (arrows), similar to what has been observed in other sequentially segmenting species. The position of these stripes suggests that *Hh*-*eve* is expressed segmentally in *H. halys* embryos, although further experiments, using double staining to definitively determine stripe register, are needed to confirm this.

To establish immunohistochemical techniques in whole-mount *H. halys* embryos, we used a monoclonal antibody raised against *Dm*-En (MAb 4D9; Developmental Studies Hybridoma Bank) that has proven to be a useful tool to examine En protein in diverse species as it recognizes an epitope located in the variable region of the homeodomain of En and Inv proteins, and does not cross-react with other homeodomain proteins [35, 36]. As shown in Fig. 1b, En protein was detected in stripes at the boundary of each segment, as expected and in keeping with the RNA pattern (Fig. 1a).

Together, these experiments establish methods to examine spatiotemporal patterns of gene expression in *H. halys* embryos. Embryo collection and fixation protocols were successfully established for *H. halys* embryos, and both antibody staining and in situ hybridization were carried out successfully. These methods are now available to examine the expression of additional genes.

### RNA interference is effective in *H. halys*

RNAi is a useful method to knock down gene expression in both plants and animals. To determine whether RNAi can be an effective tool for *H. halys*, we tested whether injection of dsRNA into adult females caused defects in offspring, so-called parental RNAi (pRNAi), which has been shown to be effective in *T. castaneum*, *D. maculatus*, *O. fasciatus* and several other insect species [50–52]. To test this method, we sought a gene whose perturbation would result in very specific morphological defects, as opposed to general lethality, so that we could clearly assess the impacts of gene knockdown. For this purpose, we chose the homeotic gene *Sex combs reduced* (*Scr*). *Scr*, like other homeotic genes, is responsible for determining segmental identity in early embryos [53]. Inappropriate expression of homeotic genes result in transformation of one body part toward another body part and loss of homeotic function results in embryonic death (reviewed in [54]). Knockdown of *Scr* by RNAi was shown to have very clear and unique effects—the transformation of proboscis toward leg—in *O. fasciatus* ([55] and reproduced by us, Additional file 3: Figure 3), and in the American cockroach, *Periplaneta americana* [24]. To test RNAi in *H. halys*, a 327-bp *Hh*-*Scr* sequence, including 51 bp of the homeobox and 276 bp upstream, isolated by degenerate PCR, was extended based on transcriptome data to an 816-bp sequence including 76 bp of 5′UTR and a coding region of 246 amino acids that includes the YPWM motif and part of the homeodomain. The homeodomain is 100% identical to a partial *Scr* gene (185 amino acids) that was isolated from the southern green stink bug (*Nezara viridula*) [56]. The partial homeodomain has an *Scr* signature sequence at the N-terminal arm of the homeodomain (highlighted in Additional file 2: Figure 2c) and is 100% identical to that of *D. melanogaster Scr*.

**Fig. 2:**
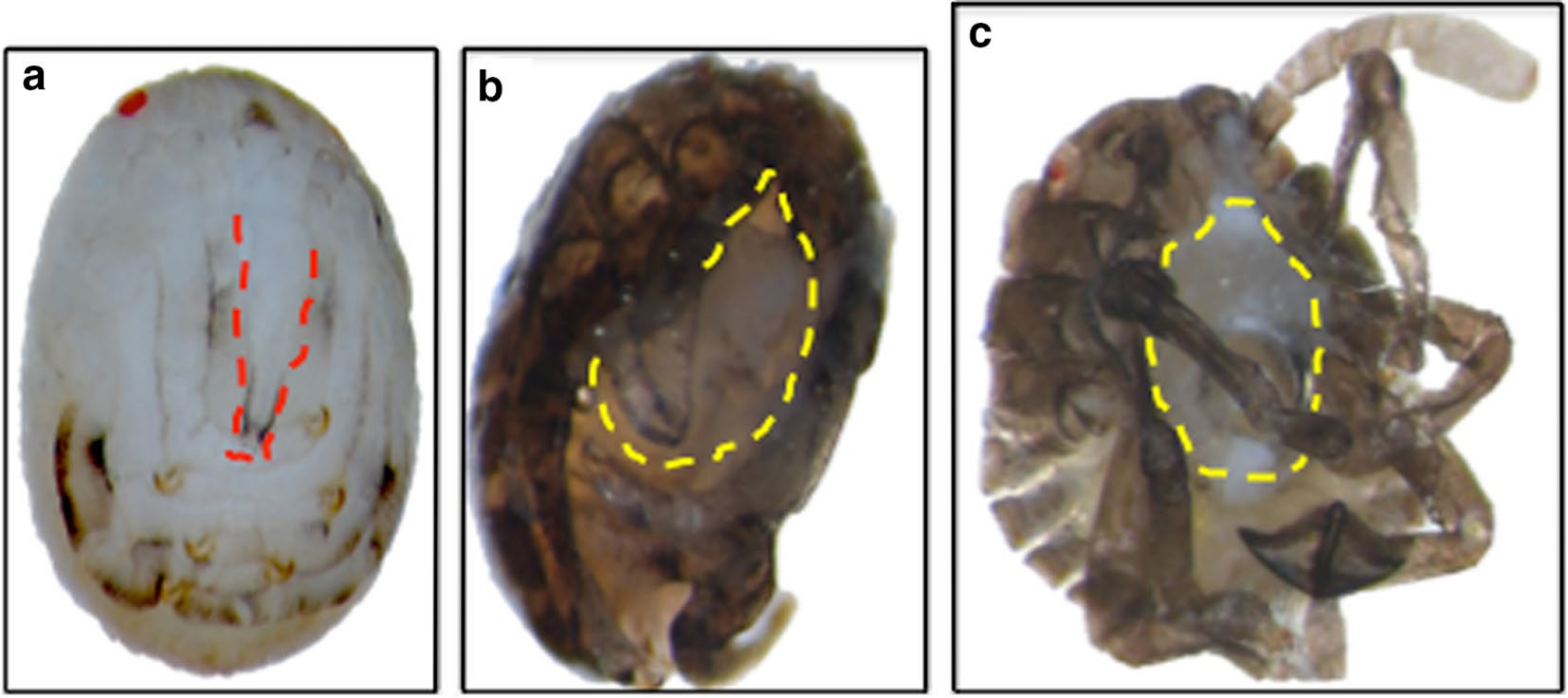
Severe defects in unhatched offspring after Scr RNAi. Photographs of nymphs dissected out of egg cases are shown. **a** Control, *gfp* ^pRNAi^ is fully developed with proboscis (red dashed line). Note that wild-type animals darken after they hatch. **b**, **c** Different examples of *Hh*-*Scr* ^pRNAi^ offspring with severely malformed or absent head structures (yellow dashed lines). These *Hh*-*Scr* ^pRNAi^ embryos were presumably unable to hatch and therefore were still within the egg shell as they matured and darkened

As expected for RNAi, a range of defects was observed in *Hh*-*Scr* pRNAi offspring. In the most severely affected embryos, death prior to hatching was observed. To examine defects, embryos were dissected out of unhatched eggs from *Scr* dsRNA-injected females. Many of these eggs were not fully developed, suggesting lethal effects of loss of *Scr* function in early embryogenesis. Some nearly hatched nymphs that were possible to remove from the egg cases lacked whole mouthparts (Fig. 2b, c).

**Fig. 3:**
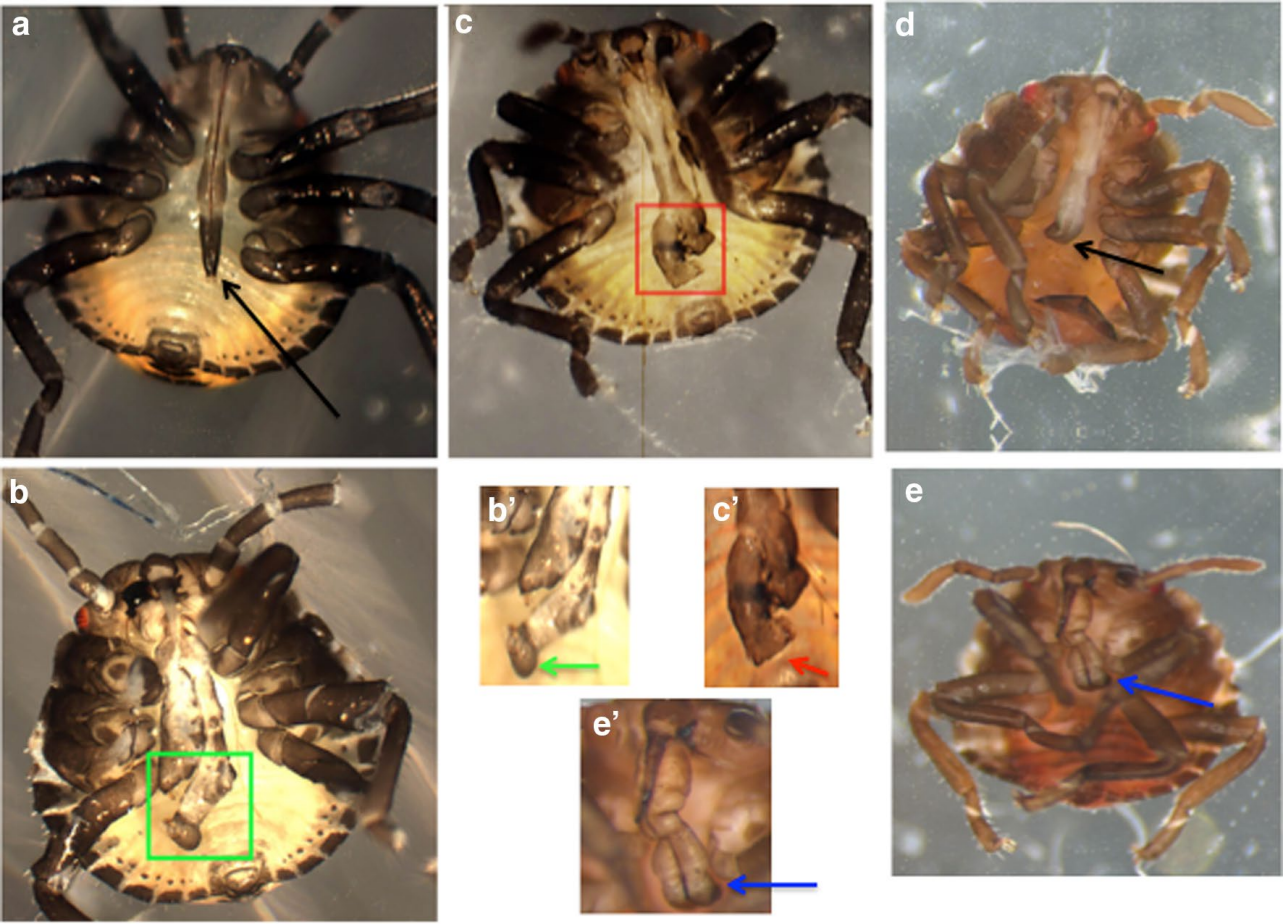
Knockdown of *Scr* by RNAi produces abnormal mouthparts in *H. halys*. Photographs of first instar nymphs are shown. **a** Wild type. The proboscis has a needle-like shape with pointed tip (black arrow). **b**–**g** Offspring of females injected with *Hh*-*Scr* dsRNA (*Scr* pRNAi). **b** First instar nymph with severe effects has a bifurcated proboscis (green square); **b**′ tip of the proboscis from panel (**b**) (green arrow). **c** First instar nymph has a blunt-ended proboscis (red square); **c**′ tip of the proboscis from panel (**c**) (red arrow), **d** another example of a transformed proboscis with bent, abnormal tip (black arrow). **e** An example of a first instar nymph showing duplication and thickening at the tip of the proboscis (blue arrow). **e**′ Enlargement of region from panel (**e**) (blue arrow)

Additional offspring of *Scr* dsRNA-injected females were observed with abnormalities indicative of homeotic transformation of the mouthparts (Fig. 3). The proboscis of wild-type first instar nymphs is needle-like in shape and has a sharp tip (Fig. 3a, black arrow). For offspring of *Scr* dsRNA-injected females, severely affected first instar nymphs had a bifurcated proboscis, and claws were seen at the tip of the proboscis (data not shown), suggestive of homeotic transformation toward leg (Fig. 3b). Some first instar nymphs had less severe defects; for example, in some cases, instead of being bifurcated, the end of the proboscises expanded into a clubbed shape (Fig. 3c). Others had shorter or bent and twisted proboscises, with partial duplication or bifurcation of distal structures (Fig. 3d, e). Finally, other nymphs hatched with normal proboscises but with smaller and withered bodies.

Overall, while the defects observed were quite specific, the penetrance of defects varied in different experiments. In some cases, all fully-developed nymphs showed abnormal mouthparts. In other cases, while increased failure to hatch was observed, hatched larvae did not appear to have mouthpart abnormalities. In addition, in any one experiment, the severity of the defects attenuated as time went on, as was also observed for *O. fasciatus* and *D. maculatus* RNAi [13, 57]. For example, embryos laid within the first 2 weeks after injection showed the most severe phenotypes (bifurcated proboscises); slightly later, embryos showed less severe phenotypes (clubbed shape proboscises); by the third week, embryos were all normal. In sum, RNAi was clearly effective in knocking down gene function in *H. halys*.

### Laboratory culture of *H. halys*

**Fig. 4:**
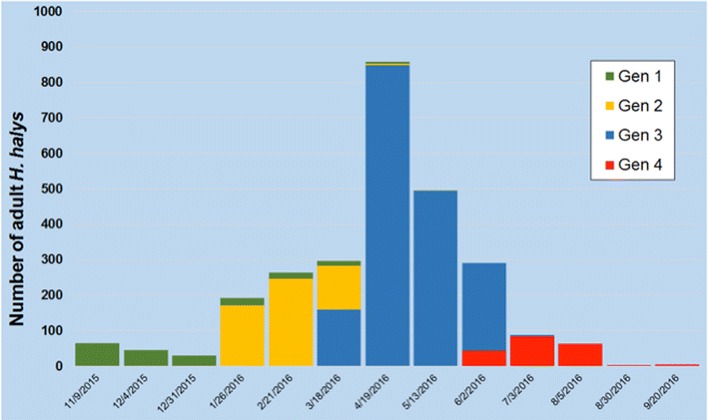
Long-term rearing of *H. halys* in culture. The number of *H. halys* adults in our lab colony is shown over time. From the egg masses obtained from Dr. Don Weber’s laboratory, 64 adults emerged (generation 1). A steady increase in adults was seen in generation 2, as expected. About 2 months later, generation 3 reached its peak at 849 adults. Following this point, our lab population experienced severe decline, with generation 4 peaking at 84 adults. This decimation was accompanied by adults displaying malformed wings and small body size

Long-term rearing of laboratory cultures of *H. halys* has been reported by others but was extremely challenging in our hands. Following two different rearing regimes (see “Methods”), one of which has been successful for multiple generations elsewhere [27, 58], we failed to maintain a stable colony without supplementation from field-caught bugs for more than three generations (Fig. 4). While we were unable to determine the cause of this colony collapse, potential causes include inbreeding depression, infection, and/or loss of symbionts over time [26]. This finding hampers experimentation because it limits the number of eggs that can be collected and the need for supplementation from outside increases heterozygosity within the colony. However, *H. halys* can be stored long term in diapause and will breed actively after this, allowing supplementation from a single wild-caught population, that in some places are so dense that it is possible to collect thousands of individuals at peak season [59]. In addition, fresh *H. halys* from different geographical regions can be caught during the spring and summer, as well as in the early fall when they congregate in human-made structures. Thus, with one generation every < 2 months, it is possible to expand colonies during the active seasons and maintain them through the winter.

These endeavors raise questions about what defines and what is necessary for the establishment of a species as a new, effective ‘model system,’ a topic that has been discussed in recent literature [60–64]. The most well-developed, traditional animal systems—flies, worms, mice—share features such as stable laboratory culture, rapid life cycles, history of genetic tools, and large amounts of genomic information, including well-annotated genomes, transcriptomes, ChIP data, publically available stocks, and more. These species form the backbone, or serve as reference points, for research in their relative phyla and beyond. However, we take the view that for comparative studies, the level of sophistication seen for these systems is not required. Techniques necessary to answer specific questions can be developed for individual species without the labor-intensive, long-term investments that have been made by communities of researchers for flies, worms and mice. This will allow for broader explorations of mechanistic diversity within realistic time frames. For *H. haly*, transcriptome data have been published [28, 58], genome sequencing is in progress [18], and we have developed the molecular tools to make use of its important point in insect phylogeny. The evo-devo field has benefited enormously from studies of seasonally collected species including, to name only a few, ctenophores [65, 66], hemichordates (e.g., [67]; echinoderms [68]; cephalochordates [69]; the centipede, *Strigamia maritima* [70], or the onychophoran *Euperipatoides kanangrensis* [71] and many others, for which long-term laboratory culture has been unsuccessful or is simply not practical. Although one advantage of insect systems is the ease with which they are cultured in the laboratory, this thinking may reflect a *D. melanogaster*-centric bias that could have the unexpected negative consequence of limiting studies of mechanistic biodiversity.

## Conclusions

We and others have taken advantage of molecular genetic techniques to analyze gene expression and function in diverse insect species. Here we present methods to analyze the expression patterns of genes in developing embryos (Figs. 1) and to study gene function by RNAi (Figs. 2, 3) in *H. halys*. The methods presented will allow other researchers in the field to expand studies of basic genetics and biology of *H. halys.* In addition, the establishment of RNAi in this species opens up the possibility of using this as a strategy for controlling *H. halys* pests in the field, an approach of increasing interest in recent years (reviewed in [72, 73]). As mentioned above, Hemiptera is a large insect order, closely related to the holometabolous insects. Here we have presented what is, to our knowledge, the first set of molecular methods to study development of a representative of the stink bug family Pentatomidae, a large family within the superfamily Pentatomoidea, which includes ~ 7000 species. Future comparative analysis will provide insight into the novel and derived regulatory mechanisms in this large clade.

## Additional files

**Additional file 1: Figure** **1.** Injection of dsRNA into *H. halys* adult females. dsRNA was injected into the abdomen of a female *H. halys* with a Hamilton syringe. The injection point is located between the 3r^d^ and 4t^h^ abdominal sterna, away from the ventral midline. The tip of the needle is held directly above the point of injection.

**Additional file 2: Figure** **2.** Conceptual translation and sequence comparison for *H. halys* genes isolated in this study. Translations of the isolated *H. halys* sequences are highlighted in yellow in each alignment. (A) Amino acid alignments for *Hh*-En and Inv. The isolated *H. halys* sequence does not encode the RS-motif which is characteristic of holometabolous Inv proteins (arrowhead). (B) Alignment of the homeodomain of several Eve proteins. (C) A cDNA encoding a 246 amino acid portion of *Hh*-*Scr*, including part of the homeodomain, was isolated. Scr amino acid alignment shows strong conservation around the YPWM motif and the homeodomain. The homeodomain N-terminal Scr signature sequence is highlighted in blue [74]. Species abbreviations are as follows: Bmor: *Bombyx mori*; Dmel: *Drosophila melanogaster*; Gbim:*Gryllus bimaculatus*; Hhal: *Halyomorpha halys*; Ofas: *O. fasciatus fasciatus;* Pame: *Periplaneta americana;* Sgre: *Schistocerca gregaria*; Tcas: *Tribolium castaneum*.

**Additional file 3: Figure** **3.** Knockdown of *Scr* in *O. fasciatus*. Scr was chosen as a standard to test RNAi in *H. halys* because of the clear phenotype observed in *O. fasciatus* [15]. These results were repeated in our lab for comparison to *H. halys*. Photos of 1s^t^ instar nymphs are shown. (A) Wild type. The proboscis has a needle-like shape with pointed tip; (B,C) Offspring of females injected with *Of*-*Scr* dsRNA. (B) 1s^t^ instar nymph with severe effects has a bifurcated proboscis (red square); (C) 1s^t^ instar nymph with less severe phenotype has a duplication at the end of the proboscis (red arrow).
