# Supplementary figures and images for: Establishment of molecular genetic approaches to study gene expression and function in an invasive hemipteran, Halyomorpha halys
Source: EvoDevo. 2017 Oct 18;8:15. doi: 10.1186/s13227-017-0078-6 (PMC5648497; doi:10.1186/s13227-017-0078-6)

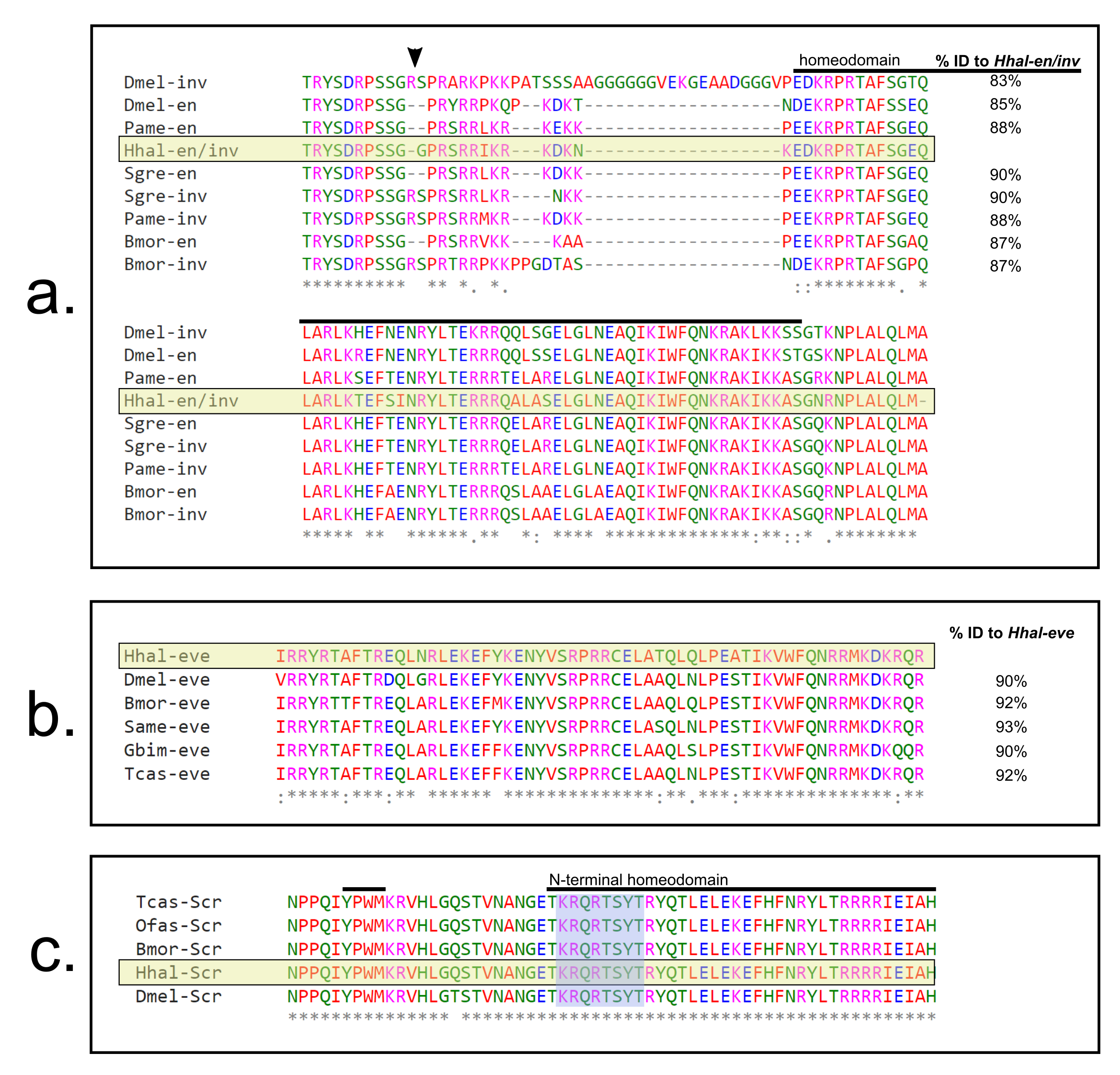

Supplement: Supplementary file 2 — Additional file 2: Figure 2. Conceptual translation and sequence comparison for H. halys genes isolated in this study. Translations of the isolated H. halys sequences are highlighted in yellow in each alignment. (A) Amino acid alignments for Hh-En and Inv. The isolated H. halys sequence does not encode the RS-motif which is characteristic of holometabolous Inv proteins (arrowhead). (B) Alignment of the homeodomain of several Eve proteins. (C) A cDNA encoding a 246 amino acid portion of Hh-Scr, including part of the homeodomain, was isolated. Scr amino acid alignment shows strong conservation around the YPWM motif and the homeodomain. The homeodomain N-terminal Scr signature sequence is highlighted in blue [74]. Species abbreviations are as follows: Bmor: Bombyx mori; Dmel: Drosophila melanogaster; Gbim:Gryllus bimaculatus; Hhal: Halyomorpha halys; Ofas: O. fasciatus fasciatus; Pame: Periplaneta americana; Sgre: Schistocerca gregaria; Tcas: Tribolium castaneum. [file 13227_2017_78_MOESM2_ESM.png]

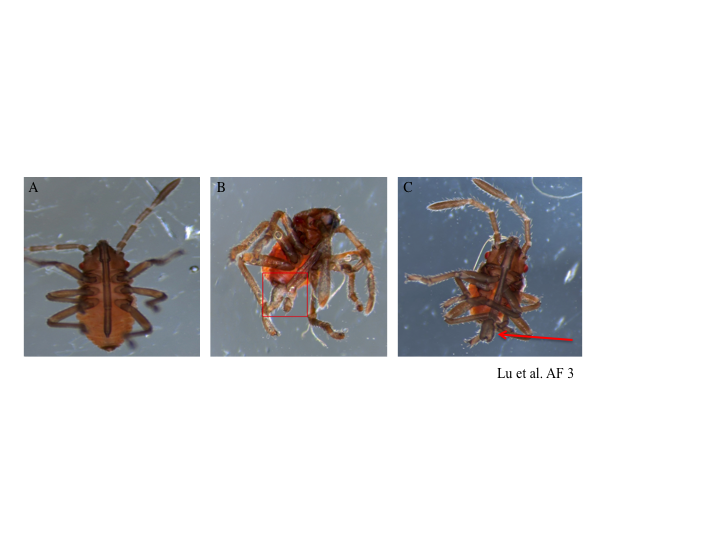

Supplement: Supplementary file 3 — Additional file 3: Figure 3. Knockdown of Scr in O. fasciatus. Scr was chosen as a standard to test RNAi in H. halys because of the clear phenotype observed in O. fasciatus [15]. These results were repeated in our lab for comparison to H. halys. Photos of 1st instar nymphs are shown. (A) Wild type. The proboscis has a needle-like shape with pointed tip; (B,C) Offspring of females injected with Of-Scr dsRNA. (B) 1st instar nymph with severe effects has a bifurcated proboscis (red square); (C) 1st instar nymph with less severe phenotype has a duplication at the end of the proboscis (red arrow). [file 13227_2017_78_MOESM3_ESM.tiff]
